# RNA-Based Technologies for Engineering Plant Virus Resistance

**DOI:** 10.3390/plants10010082

**Published:** 2021-01-02

**Authors:** Michael Taliansky, Viktoria Samarskaya, Sergey K. Zavriev, Igor Fesenko, Natalia O. Kalinina, Andrew J. Love

**Affiliations:** 1 Shemyakin-Ovchinnikov Institute of Bioorganic Chemistry of the Russian Academy of Sciences, 117997 Moscow, Russia; viktoriya.samarskaya2012@yandex.ru (V.S.); szavriev@ibch.ru (S.K.Z.); fesigor@gmail.com (I.F.); kalinina@belozersky.msu.ru (N.O.K.); 2 The James Hutton Institute, Invergowrie, Dundee DD2 5DA, UK; 3 Belozersky Institute of Physico-Chemical Biology, Lomonosov Moscow State University, Leninskie Gory, 119991 Moscow, Russia

**Keywords:** dsRNA, siRNA, miRNA, lncRNA, amiRNA, tasiRNA, RNAi, CRISPR-Cas

## Abstract

In recent years, non-coding RNAs (ncRNAs) have gained unprecedented attention as new and crucial players in the regulation of numerous cellular processes and disease responses. In this review, we describe how diverse ncRNAs, including both small RNAs and long ncRNAs, may be used to engineer resistance against plant viruses. We discuss how double-stranded RNAs and small RNAs, such as artificial microRNAs and trans-acting small interfering RNAs, either produced in transgenic plants or delivered exogenously to non-transgenic plants, may constitute powerful RNA interference (RNAi)-based technology that can be exploited to control plant viruses. Additionally, we describe how RNA guided CRISPR-CAS gene-editing systems have been deployed to inhibit plant virus infections, and we provide a comparative analysis of RNAi approaches and CRISPR-Cas technology. The two main strategies for engineering virus resistance are also discussed, including direct targeting of viral DNA or RNA, or inactivation of plant host susceptibility genes. We also elaborate on the challenges that need to be overcome before such technologies can be broadly exploited for crop protection against viruses.

## 1. Introduction

Due to climate change and alterations in cropping systems, plant viral disease outbreaks will likely have increasingly negative impacts on food production, which will bring new challenges to current agricultural practices worldwide. To advance virus disease management, rapid, efficient, and safe responses are required to minimize viral threats. The use of virus-resistant crop varieties is a traditional and efficient way to reduce losses caused by viral infections. However, conventional antiviral breeding strategies, even augmented with modern molecular techniques such as quantitative trait locus (QTL) mapping, marker-assisted selection, and whole-genome sequence-based approaches, are slow and laborious and require monitoring of large crop populations over multiple generations [1].

Advances in genetic engineering and plant transformation techniques in the early 1980s enabled the rapid production of virus-resistant transgenic plants [2]. Since then, many research groups have exploited this approach and generated resistance against numerous viruses in many different crops via transgenic expression of various virus-derived or non-viral genes or their fragments [2].

It should be noted that historically, mainstream approaches to engineering plant virus resistance have been driven by a classical view of the central dogma of molecular biology, in which the genetic information contained in DNA is transcribed into mRNA which in turn directs the synthesis of particular proteins, and variations in proteins give rise to different traits. Accordingly, many transgenic plants were generated to express virus-encoded proteins (to potentially interfere with viral functions), with the expectation that any resistance obtained would be protein-mediated. However, in many cases, the resistance had been surprisingly proven to be RNA-mediated. This phenomenon was named RNA silencing or RNA interference (RNAi) [3]. This is a eukaryotic sequence-specific mechanism that controls endogenous gene expression and destroys foreign nucleic acids. RNAi is triggered by the presence of double-stranded RNA (dsRNA) precursors derived from either host plant hairpin RNA structures or viral replicative dsRNA intermediates, which are cleaved into small (18-27 nucleotides, nt) non-coding (nc) interfering RNAs (siRNAs) or micro RNAs (miRNAs), which are associated with some plant proteins to further mediate the amplified sequence-specific inactivation/degradation of foreign (e.g., viral) or endogenous RNAs respectively [4,5,6]. Importantly, siRNAs operate as defenders that directly target their own (invasive) progenitors, whereas miRNAs silence their endogenous gene targets, many of which are involved in developmental processes and stress responses.

While the small siRNAs and miRNAs constitute important classes of regulatory ncRNA, many other types of ncRNAs are known, and all of these form the majority of the transcriptome, given that only about 2% of transcribed RNAs encode proteins [7]. ncRNAs also include well-known classes of RNAs involved in translation such as tRNAs and rRNA, small nuclear RNAs (snRNAs) involved in RNA splicing, and small nucleolar RNAs (snoRNAs) mostly involved in the regulation of posttranscriptional modification of rRNAs [8]. In addition, the plant genome encodes tens of thousands of long ncRNAs (lncRNAs; >200 nt in length) which include linear and circular lncRNAs with no or little coding capacity [9]. Over the past decade, tens of thousands of novel lncRNAs have been annotated in animal and plant genomes. LncRNAs have emerged as highly important regulatory molecules with a role in plant growth, development, and responses to abiotic and biotic (including virus attack) stresses [9]. However, their precise molecular functions in diverse biological processes are only just starting to be elucidated.

Thus, while the central dogma of DNA>RNA>protein still holds true, ncRNAs can greatly influence this information flow and are, therefore, now an emerging major new source of next-generation targets which may be exploited in diverse applications, including conferring plant resistance against pathogens and viruses in particular [8].

RNA technologies provide us not only with new sources of resistance but also with powerful tools to manage plant defense responses. With regards to antiviral resistance, RNAi-based approaches have long been used in the form of transgene-induced RNA silencing to target the degradation of diverse viral RNAs or to inactivate virus susceptibility genes in various crops [2,10,11,12]. However, more recently, exogenous applications of dsRNAs, siRNAs, and hairpin RNAs have been developed and exploited to trigger viral RNA degradation, which could be considered more sustainable, safe, and publicly acceptable than transgenic technology [13]. Another breakthrough has come with the development of RNA-guided CRISPR-Cas genome editing technologies which have been applied in plant virology [14,15,16]. CRISPR-Cas or clustered regularly interspaced short palindromic repeats (CRISPR)-CRISPR-associated genes (Cas) is a prokaryotic defense system that evolved to eliminate foreign plasmids or viral DNA and has been reprogrammed into genome editing technology in eukaryotic organisms (reviewed in [17]). The CRISPR-Cas genome editing system includes a Cas endonuclease and a single-guide RNA (sgRNA). The sgRNA contains a targeting sequence of approximately 20 nt which defines its specificity and a scaffold for Cas protein binding. The Cas protein guided by sgRNA targeting creates a double-strand break at the target DNA site which is then repaired by cellular machinery. This event is often accompanied by deletions or insertions in the cleaved region of the open reading frame (ORF), which leads to knockout of the target gene. RNA-targeting Cas endonucleases have also been described [17,18,19] and successfully used for engineering plant virus resistance in a way similar to RNAi: either by direct targeting of viral DNA or RNA or by inactivation of plant host genes required for the development of virus infections (reviewed in [14,15,16]. The use of various sequences and the mechanisms of RNA-based engineering plant virus resistance have been considered in detail in a number of reviews [13,14,15,16,20,21,22,23,24,25,26,27,28,29]. However, there has not been a review that has covered all of the approaches used to date. In this review, we will address this gap and discuss different versions of RNA technologies as well as their advantages and drawbacks. The exciting progress in the development of the RNA technologies for engineering virus resistance in plants also raises certain technical and societal challenges that need to be addressed before these technologies can enter agricultural practice: these will be outlined below.

## 2. Non-Coding RNAs and Plant Antiviral Defense

### 2.1. siRNAs

Of the different types of ncRNAs, siRNAs have attracted considerable attention due to their direct targeting of viral RNAs for degradation as a part of the natural RNAi-based antiviral immune response [4,30]. For RNA-containing plant viruses, the formation of siRNA is initiated by the Dicer-like enzyme (DCL), which cuts virus-specific dsRNAs (which often arise as intermediates in virus replication) into short siRNA fragments of 21–25 nt. One of the two chains (guide strand) of each duplex RNA fragment is then incorporated into a multi-protein RNA-induced silencing complex (RISC) which includes Argonaute (AGO), an endonuclease. This complex facilitates the pairing of the guide strand with a complementary sequence in viral RNAs which is then cleaved by the AGO component [4,5,31,32,33,34]. The resulting “aberrant” viral RNA cleavage products are amplified by endogenous plant RNA-dependent RNA polymerases (*RdRp*), and serve as secondary siRNAs that systemically spread throughout the plant; which is crucial for efficient RNAi-based antiviral defense [35]. RNA silencing may further be enhanced via methylation of the siRNAs by the small RNA methyltransferase HUA ENHANCER1 (HEN1) [34], which serves to improve the silencing signal by promoting siRNA stability and longevity.

For DNA-containing plant viruses (e.g., geminiviruses) the precursors of siRNAs are presumably produced by DNA-dependent RNA polymerase II (Pol II) which mediates bidirectional transcription in both sense and antisense orientations on the viral DNA. Such transcripts can pair virus-specific mRNAs and hence form perfect dsRNAs to be processed by some DCLs into siRNAs [36].

To successfully overcome siRNA defenses and infect plants, viruses have evolved suppressors of RNA silencing (VSRs) which may target key steps in the siRNA pathways by inhibiting siRNA production, sequestering siRNAs, or preventing the spread of RNA silencing signals [37]. Some other viruses such as red clover necrotic mosaic virus may evade silencing by adopting viral RNA structures that are incompatible with the host plant siRNA potentiation machinery [38].

Thus, siRNA-triggered RNAi represents a robust host defense mechanism against plant viruses which has been proven for numerous viruses in many plant species and crops [13,20]. With increasing knowledge of this mechanism, siRNA-based technologies have been emerging as an innovative approach with which to control plant viruses in practical agriculture.

### 2.2. miRNAs

Fundamentally, miRNAs and siRNAs share some similarities in size, structure, and functions. Both are short duplex RNA molecules that may exert gene silencing effects at the RNA level and are derived from double-stranded regions of RNA precursors. They both require DCL and AGO proteins for processing, maturation, and action, although the species of DCL and AGO may be different. However, there are many important differences between miRNAs and siRNAs. A major distinction between them is that siRNAs are processed from foreign dsRNAs (such as those derived from transposons, transgenes, or viruses) whereas miRNAs are derived from endogenous precursor transcripts containing double-stranded (typically hairpin) regions encoded by miRNA genes (MIR genes). miRNAs are derived from longer, primary transcripts termed pri-miRNAs. The pri-miRNAs contain an RNA hairpin in which one of the two strands includes the mature miRNA fragment. This hairpin then is cut out by a microprocessor enzyme complex which includes DCL1 nuclease (a member of an RNase III endonuclease family) and HYL1, a dsRNA binding protein [21,39], which act to generate precursor miRNA (pre-miRNA). The pre-miRNA is further cleaved to generate a short RNA duplex in which one strand is the mature miRNA. The mature miRNA is methylated by HEN1 and taken up by the RISC complex to further potentiate RNAi. Most plant miRNAs typically have perfect or near-perfect complementarity with their targets. This is consistent with their primary mode of action being cleavage of target mRNAs. However, some plant miRNAs and their natural targets may have several mismatches, which while they do not initiate cleavage, may promote repression of mRNA translation [39].

Many miRNAs are evolutionarily conserved across major taxa of the Kingdom *Plantae* from mosses to monocots and eudicots and can be grouped in two classes: one which is conserved and ancient, and one which consists of miRNAs that are less conserved and younger [22,40]. *In toto*, miRNAs constitute a highly diverse set of molecules which may match nearly all RNAs encoded in a plant genome, thereby controlling almost any aspect of the plant life cycle; playing key roles in growth and development, signal transduction, innate immunity, and responses to various ecological biotic and abiotic stresses [22].

With regards to virus infections, information on miRNAs has mainly focused on their role in the induction of viral disease symptoms and antiviral defense mechanisms. It is well documented that some viruses may control accumulation rates of miRNAs involved in plant symptom development [21]. For example, the severe strain of cucumber mosaic virus (CMV)—Fny suppressed the accumulation of miR159 in *Arabidopsis* plants, thus elevating the accumulation of its gene target transcripts MYB33 and MYB65 (which encode transcription factors that are involved in gibberellin signal transduction). This consequently led to the production of severe viral symptoms [41]. Similar findings were also observed with CMV-LS infection, which resulted in reduced accumulations of miR159, miR165, and miR166 which operate as negative regulators of symptom development [41]. It has also been reported that miR171 may be downregulated by rice streak virus (RSV) infection, which was implicated in reduced chlorophyll content and leaf yellowing [42]. Interestingly, while miRNAs would be expected to contain similarities with host genes and alter their expression via the RNA silencing machinery, it is also possible that siRNAs derived from viral genomes may contain occasional similarities to host genes and potentiate silencing of those genes. For example, siRNA derived from the CMV Y-satellite RNA was found to target the chlorophyll biosynthetic gene (Chll), causing Chll mRNA silencing, which induced yellowing symptoms in tobacco plants [43]. Some of the siRNAs derived from potato virus Y (PVY) are completely complementary to the gene encoding translationally controlled tumor protein (TCTP). This protein is required for successful infection of tobacco by PVY and hence, its targeting by PVY-specific siRNA effectively knocks the protein down and blocks invasion [44]. Why the virus forms siRNA that has an unfavorable impact on itself remains unclear.

Many components of the antiviral silencing machinery such as *AGO or RdRp* genes, which are involved in siRNA biogenesis and activity, are themselves natural targets of various plant miRNAs. For example, the antiviral *AGO1* is the target gene for miR168. To prevent degradation of *AGO1* transcripts and thereby alleviate *AGO1*-mediated antiviral defense, plants have evolved a decoy protein gene *AGO18* [45]. *AGO18* transcripts compete with *AGO1* transcripts for miR168 to attenuate the cleavage of *AGO1* mRNA. Expression of *AGO18* in rice was shown to be activated upon RSV infection, conferring resistance against this virus through partial miR168 sequestration [45]. miR444 has been found to promote antiviral resistance against RSV by enhancing the *RdRp1*-mediated RNA silencing pathway in rice by targeting *RdRp1*-inhibiting proteins [46].

It has also been shown that some miRNAs can target genes that trigger antiviral resistance [47]. For example, miR6019 and miR6020 target mRNA of tobacco *N* gene encoding the resistance TIR-NB-LRR protein gene that mediates hypersensitive response against tobacco mosaic virus (TMV). Overexpression of these miRNAs decreases levels of TIR-NB-LRR transcripts and attenuates *N*-mediated resistance to TMV, suggesting that miR6019 and miR6020 play an important role in controlling resistance to TMV [48,49].

General plant responses contributing to antiviral resistance are often orchestrated by the rapid production of reactive oxygen species (ROS) and several phytohormones such as salicylic acid (SA), jasmonic acid (JA), and auxin [50,51]. It has been demonstrated that ROS- and phytohormone-triggered signaling pathways can be regulated by miRNAs, and these can in turn be influenced by virus infection. For example, miR528 can negatively regulate ROS levels by cleaving L-ascorbate oxidase (AO) messenger RNA [52], thereby reducing AO-dependent accumulation of ROS. Upon RSV infection, miR528 is sequestered by *AGO18*, resulting in increased AO activity and elevated ROS accumulation; rendering plants more resistant to the virus [52,53]. miR319 which reduces expression of target gene *TCP21* and JA concentrations in rice had elevated abundance after infection by rice ragged stunt virus, leading to a more virus-susceptible phenotype [54]. Recently, transgenic overexpression of mutant miR393 in rice was shown to confer higher susceptibility to rice black-streaked dwarf virus infection (RBSDV), by suppressing the auxin receptor TIR1; suggesting that auxin signaling plays an important role against RBSDV infection in rice [55].

As exemplified by these case studies, miRNAs are currently regarded among the most important gene regulators. As discussed, these small RNAs are involved in many pivotal aspects of plant-virus interactions and are emerging as the next generation targets for engineering plant virus resistance.

### 2.3. LncRNAs

Another group of non-coding RNAs playing important roles in many biological processes is lncRNAs. LncRNAs are defined as transcripts longer than 200 nt with little or no coding potential. Similar to coding mRNAs, most lncRNAs are transcribed by Pol II. They may also have typical mRNA-like structures such as 5′m^7^ G cap, 3′ poly (A) tail, and exon-exon junctions [7,9,56]. In addition, the two plant-specific RNA polymerases—Pol IV and Pol V—can produce non-polyadenylated lncRNAs and hundreds of such transcripts were induced in *Arabidopsis* under stress conditions [57,58]. In general, lncRNAs are typically less sequence conserved and abundant than mRNAs but have greater tissue specificity.

Based on genomic origin, lncRNAs can be classified as intergenic, intronic, or exonic regions in sense or antisense orientation [59]. Besides these widely discussed lncRNAs types, short-lived medium-length lncRNAs (from 200 to 2000 nt), such as those derived from promoter upstream transcripts (PROMPTs) and enhancer RNAs (eRNAs) were also identified in plant and animal cells [60,61]. Although most functional plant lncRNAs have not yet been well characterized, they have been implicated in multiple mechanisms to control a diverse range of gene expression pathways [7,9,56]. LncRNAs may regulate the expression of the genes present on the same locus in *cis* and that of distant genes in *trans. Cis*–acting regulatory lncRNAs modulate the expression of the neighboring target genes either by recruitment of Pol II or through the event of getting transcribed, which results in local chromatin conformational changes that facilitate downstream mRNA transcription [7].

*Trans*-acting lncRNAs may act as scaffolds to facilitate the recruitment of protein(s) and the formation of RNA protein complexes. Such interactions are usually mediated by specific lncRNA domains and may regulate gene expression either through sequestering proteins and preventing them from reaching their target (as a decoy) or by delivering them to specific target sites (as a guide) [7,9]. LncRNAs may also bind miRNAs (as target mimics) to sequester miRNAs, which disrupts their activity to silence specific mRNAs. Another emerging function of lncRNAs is to control the epigenetic state of particular genes by regulating DNA and histone methylation [62]. For example, certain lncRNA can guide DNA methyltransferase-containing complexes to target genomic loci for methylation and transcriptional repression. In contrast, some other lncRNAs recruit histone methyltransferases to activate gene expression by promoting histone methylation. Depending on the mode of action in the transcriptional or posttranscriptional control of gene expression, lncRNAs can reside either in the nucleus or cytosol [63]. The subcellular localization of lncRNAs is controlled via intrinsic RNA sequence localization elements and/or through their interaction with different binding proteins.

The repertoire of biological processes and mechanisms of action of the lncRNAs are rapidly growing. Plant lncRNAs are known to play pivotal roles in the regulation of flowering time, modulation of reproductive organ development, leaf development, auxin signaling, photomorphogenesis, and responses to biotic and abiotic stresses [63].

It is conceivable that lncRNAs may also play important roles in plant-virus interactions. Indeed, it has been shown that both RNA-containing tobacco rattle virus [64] and DNA-containing tomato yellow leaf curl virus (TYLCV) [65,66] significantly change the pattern of lncRNA accumulation upon virus infection. Moreover, some tomato lncRNAs have been shown to act as competing endogenous target mimics for miRNAs in response to TYLCV, suggesting a role in the regulation of virus resistance in the tomato [65].

The ELENA1 lncRNA identified in another recent study was shown to take part in controlling plant defense by modulating the SA pathway [67]. It was found that ELENA1 is able to interact with fibrillarin, the major protein of the nucleolus which also acts as a negative regulator of plant immunity [68,69]. This interaction with ELENA1 evicts fibrillarin from a complex with MED19a, rendering the MED19a active, which subsequently drives the transcription of SA-inducible immune—responsive (pathogenesis-related) genes [67]. Taken into account that fibrillarin has also been implicated in interactions with plant viruses [68,69], we can anticipate that ELENA1 and/or some other lncRNAS, may play a regulatory role in controlling plant antiviral defense responses.

Interestingly, the citrus Tristeza virus (CTV) encodes its own subgenomic lncRNA, LMT1 [70]. Infection of *Nicotiana benthamiana* with a CTV isolate deficient in LMT1 production (CTV-LMT1d) led to an elevated SA accumulation (which typically enhances virus resistance [71] and a consistent reduction in susceptibility to the virus in comparison to the wild type virus [70]. This study also reported that ectopic expression of LMT1 RNA could suppress SA accumulation and subvert the low-infectivity phenotype of CTV-LMT1d; suggesting that LMT1 promotes plant immune evasion by suppressing SA accumulation [70]. Further studies are warranted to elucidate mechanisms underlying the role of lncRNAs in plant virus infections.

Circular RNAs (circRNAs) are a group of endogenous non-coding ssRNAs that have a closed-loop structure [72]. CircRNAs are generated in the back-splicing process from pre-mRNAs, in which the 5′- and 3′-ends are joined by covalent bonds. Typically, circRNAs exhibit a much higher degree of conservation than linear lncRNAs, but their abundance is low. Due to the existence of similar miRNA binding sites in both circRNAs and mRNAs, many circRNAs may interfere with miRNA-mRNA interactions; indicative of them playing a broad regulatory role in various processes in plants such as growth, development, reproductive processes, biotic and abiotic stress responses [72]. Expression of circRNAs in plants is often promoted by different ecological stresses such as heat, drought, chilling, or pathogen attack. With regards to virus infections, circRNAs have been predicted to function as negative regulators of defense responses to TYLCV infection in tomato [65] and maize Iranian mosaic virus infection in maize. Interestingly, viroids and some viral satellite RNAs are also single-stranded circular RNAs; but in contrast to host circRNAs, they are replicative and infectious or associated with virus infections (able to spread from cell-to-cell as well as from plant to plant [73].

### 2.4. Small Peptides in lncRNAs

Despite their definition as non-coding RNAs, a growing number of reports have suggested the existence of stably expressed and functional peptides (or microproteins) translated from lncRNAs [74]. These peptides encoded by small open reading frames (smORFs-encoded peptides [SEPs]) are shown to regulate a diverse range of cellular processes in plants and animals [75,76]. Various computational tools and experimental approaches such as ribosomal profiling and mass-spectrometry analysis have recently been explored and exploited to differentiate between coding and non-coding RNAs and to identify ORFs that encode SEPs [74,77]. Besides lncRNAs, pri-miRNAs may also encode SEPs (miPEPs), including miPEP165a from *Arabidopsis,* miPEP171b from *Medicago* [78], and miPEP156a which is evolutionarily conserved in *Brassicaceae* [79]. These peptides presumably modulate the expression of their corresponding miRNAs and activate target genes responsible for tissue and organ development.

There is a growing body of evidence that SEPs play an important role in animal cell immunity [80], suggesting the existence of SEPs with similar functionality in plants. Although the role of SEPs produced by various lncRNAs in plant-virus interactions has not been investigated so far, this function remains a distinct possibility given that novel peptide-based regulators were discovered in the cluster of lncRNAs/circRNAs, which control the animal antiviral response [81]. Future studies on the effect of such small peptides on plant-virus interactions would present a challenging task.

### 2.5. Other Non-Coding RNAs

As mentioned above, other classes of non-coding RNAs include rRNAs, tRNAs, snRNAs, and snoRNAs. There are no doubts that some functional crosstalk may exist between various activities of these RNAs and virus infections. A striking example of such interplay is that sophisticated functional mimics of tRNAs (transfer RNA-like structures) are found at the 3′-ends of the genomes of some plant positive-strand RNA viruses [82], which may compete for host tRNA binding factors. Viral RNAs may also compete with rRNAs for some ribosomal proteins [83]. These observations may contribute to a better understanding of mechanisms underlying plant-virus interactions and provide new platforms for virus control. However, at present, the involvement of tRNAs, rRNAs, snRNAs, and snoRNAs as targets in plant antiviral responses with RPs is largely unknown. Therefore, exploring antiviral strategies based on the use of these RNA classes is outside the scope of this review article and will likely be the focus of future research.

## 3. RNA-Based Tools for Engineering Viral Resistance

### 3.1. Host Induced Gene Silencing

**Figure 1:**
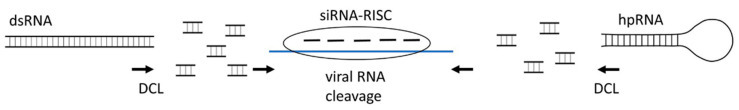
The RNAi antiviral pathway is induced by dsRNA or hpRNA. dsRNA and hpRNA are recognized by plant DCL proteins which then cleave them into siRNAs. siRNAs are subsequently incorporated into the RISC, which guides sequence-specific degradation of viral RNAs or mRNAs encoding virus susceptibility proteins. dsRNA or hpRNAs can be expressed transgenically (HIGS) or applied exogenously (SIGS).

Pioneering work published in 1986 by Powell Abel et al. [84] demonstrated that transgenic plants that were engineered to express virus-derived sequences, such as the TMV coat protein, are able to exhibit a degree of protection against infection by the virus. Numerous efforts since then to express various types of virus-encoded proteins (such as coat proteins, replicates, movement proteins, and others) have confirmed the wide applicability of this approach in conferring protection against various plant viruses (reviewed in [2]). However, in many cases, the mechanisms underlying this type of resistance were actually mediated by RNA silencing rather than by protein-based interference [2]. On a practical level, RNA-mediated protection appears to be more preferable than protein-derived resistance because it does not involve the accumulation of foreign proteins in transgenic plants and is hence more efficient and environmentally safer. Consequently, later studies to engineer transgenic virus-resistant plants have focussed on the development of RNA silencing technology. Indeed, transgenic expression of different precursors for siRNA production, such as sense or antisense RNA, hairpin RNA, or dsRNA in host plants conferred protection against virus diseases (Figure 1). This “host induced gene silencing” (HIGS) has been successfully applied to confer plant resistance to 60 different (RNA and DNA) viruses in nearly 30 plant crops, which is detailed in recent comprehensive reviews [2,13,23,24]. However, although HIGS has been demonstrated to be a very effective technology for controlling virus diseases, it is time-consuming, expensive, and still restricted by GMO regulations and poor public acceptance.

### 3.2. Exogenous dsRNA (hpRNA)-Induced Gene Silencing

To address these hurdles and public concerns, another approach termed “spray induced gene silencing” (SIGS) has been developed whereby RNA silencing-based antiviral protection is induced by spraying plants with exogenous dsRNA (or hpRNA), complementary to viral RNAs [13]. In addition, mechanical inoculation or high-pressure spraying of dsRNA/hpRNAs have also been successfully used to protect the plant from viruses. Thus far, exogenous technologies have been applied to target over 10 different economically important plant viruses in more than 10 plant species (Table 1). The efficacy of RNAi-mediated processes in reducing or delaying viral infections is dependent on what regions of different virus genomes are targeted by dsRNA. Thus, selecting the “right” target sequence is a crucial consideration for designing dsRNA for virus control.

PMMoV, pepper mild mottle virus; TEV, tobacco etch virus; AMV, alfalfa mosaic virus; PSTVd, potato spindle tuber viroid; CEV, citrus exocortis viroid; CChMVd, chrysanthemum chlorotic mottle viroid; SCMV, sugarcane mosaic virus; PRSV, papaya ringspot virus; CymMV, cymbidium mosaic virus; CMV, cucumber mosaic virus; ZYMV, zucchini yellow mosaic virus; BCMV, bean common mosaic virus; ToLCV, tomato leaf curl virus; PVY, potato virus Y; PPV, plum pox virus; PSbMV, pea seed-borne mosaic virus; LDH, layered double hydroxide clay nanosheets.

It is also worth noting that the antiviral RNAi-based resistance mechanism induced by the exogenous dsRNA may be further enhanced via pattern triggered immunity (PTI). PTI is a general plant response that is triggered by a conserved microbe—or pathogen—ssociated molecular patterns (MAMPs or PAMPs, respectively) as the first step of plant defense [102]. Recently, it has been recognized that PTI restricts virus infection in plants and that this antiviral response is mediated by dsRNA, which is a “common pattern” of virus-infected plants [102,103].

Altogether, these scientific achievements create new opportunities for practical applications of dsRNA-based approaches for crops. Recently developed breakthrough technological platforms for large-scale cell-free or bacterial production (at kilogram scale) and purification of dsRNA at as low a cost as $0.5–1/g [25], greatly enhances prospects for rapid commercialization of the biocontrol SIGS technology. However, there are still some limitations that hinder its widespread application, and these must be addressed before entering the market. Among them is the low stability of “naked” dsRNA in the environment and its poor uptake by plant cells [26]. Thus, exploring new approaches to enhance stability and uptake of dsRNAs is a prerequisite of successful commercialization as discussed in the section below.

### 3.3. Host Gene Targets for SIGS

Another approach to engineer virus resistance using SIGS is to silence virus susceptibility genes in host plants. Among the known susceptibility genes required for successful infection with some plant viruses, such as potyviruses, are those encoding the translation initiation factors eIF4E, eIF(iso)4E, and eIF4G [104]. Inactivation of a single isoform of these factors can induce resistance to a virus without compromising plant health or fitness. For example, silencing of eIF4E or eIF(iso)4E via transgenic expression of hairpin RNAs in plum plants conferred partial protection against PPV [105]. Another example of a “susceptibility” gene is a coilin-encoding gene. Coilin is a structural protein of subnuclear bodies, Cajal bodies, that have been shown to enhance susceptibility to PVY, and as a result, resistance to PVY was significantly enhanced in transgenic coilin-silenced (expressing coilin hairpin RNA) plants [106].

Although this has not yet been experimentally verified, one can suggest that exogenous dsRNA designed to silence a particular host susceptibility gene target can be applied to intact plants without the need for plant transformation. In addition, modification of individual cultivars is not required due to the sequence similarity of targeted genes; however, only a few such genes have been identified. Therefore, finding novel universal and effective targets among host genes and non-coding RNAs is a challenge for further development and marketing of the SIGS technology.

### 3.4. New RNA Tools for SIGS

**Figure 2:**
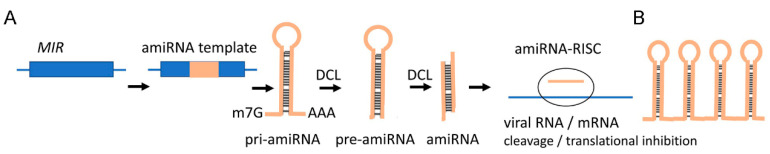
The antiviral amiRNA pathway. (**A**) amiRNA silencing constructs can be generated by replacing known miRNA sequences in a *MIR* gene with a sequence designed to target and degrade virus RNAs or host mRNAs that encode proteins conferring virus susceptibility. In transgenic plants, the amiRNA construct is transcribed by Pol II into pri-amiRNA which is sequentially processed through pre-amiRNA into amiRNA. Pri-amiRNA or pre-amiRNAs can be potentially applied exogenously and processed into amiRNA inside the cell using cellular RNAi machinery. Mature amiRNAs are incorporated into the RISC to direct the cleavage of target RNA to mediate silencing. (**B**) Multiple amiRNAs may also be easily expressed from a single precursor as tandem repeats which can target multiple sites of the same virus or several viruses.

**Figure 3:**
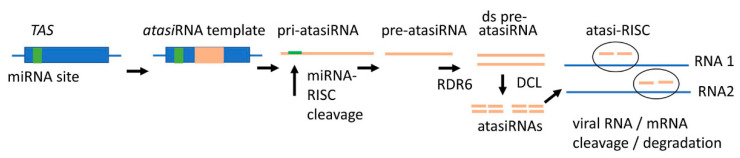
The antiviral atasiRNA pathway. The diagram schematically shows how atasiRNA silencing construct can be generated by replacing a known tasiRNA sequence in a *TAS* gene with a sequence containing four different atasiRNA fragments targeting multiple sites in two different viral or host RNAs. Like all natural *TAS* transcripts, transcripts generated from the modified *TAS* genes (pri-atasiRNAs) contain a miRNA targeting site which is required for processing pri-atasiRNAs into pre-tasiRNAs. After an initial cleavage at this site triggered by miRNA, RDR6 converts the obtained transcripts into ds pre-atasiRNA fragments which are consequently processed by DCL to generate siRNAs that target viral or host RNAs. (D)’s pre-atasi RNAs can be potentially applied exogenously.

Until now, only exogenous dsRNA has been used in SIGS as a silencing trigger. In the future, the arsenal of RNA triggers for SIGS could be significantly expanded by adding next-generation tools such as artificial miRNAs (amiRNAs), artificial trans-active siRNAs (atasiRNAs), and target mimics of several kinds [27]. A corollary is that the siRNA molecules formed during dsRNA-mediated SIGS are not predefined. Since the loading of siRNAs into the silencing effector, AGO requires certain sequence features andmany siRNAs generated from dsRNAs may not fit into AGO. Natural miRNAs are released from well-defined secondary structures in their pri-miRNA transcripts. In the amiRNA approach, mature miRNA sequences within pri-miRNAs are replaced by customized 21-nt RNA fragments that are complementary to viral targets and have favorable features for AGO loading [23]. When entering plant cells, pri-amiRNAs should be transcribed and processed into mature miRNA with the designed specificity by the host miRNA biogenesis machinery to confer virus resistance (Figure 2A,B). This concept has been validated by experiments to develop virus resistance in various transgenic crops, for example, against the tomato leaf curl New Delhi virus, CMV, RBSDV, RSV, wheat streak mosaic virus, and wheat dwarf virus (WDV) (reviewed by Cisneros and Carbonell [28]). One potential concern of this approach is that pri-amiRNAs are single-stranded molecules (with a stem-loop structure) that are more sensitive to enzymatic degradation than dsRNAs, if applied exogenously. This obstacle can be avoided by using so-called artificial transacting siRNAs (atasiRNAs). Precursors of natural tasiRNAs originate from TAS loci and are converted to perfectly paired dsRNA precursors which are more stable than the ssRNA precursors of amiRNAs, and, therefore, may be better suited for SIGS application, particularly if they could be bulk produced artificially (Figure 3). The feasibility of using atasiRNAs was confirmed by Carbonell et al. [107] using tomato spotted wilt virus atasiRNA in transgenic tomato crops. The artificial multiplexing of both amiRNA and atasiRNA precursors could potentially allow the insertion of multiple RNA fragments targeting several sites in one or more viruses.

The miRNA mimic technology (miR-Mimic) is another novel approach for gene silencing. miRNA mimics are artificially synthesized dsRNA molecules imitating miRNA duplexes. Once delivered into the cell, these RNA fragments mimic natural miRNAs which bind to the target mRNA molecules and block their expression either by degradation or by translational repression [108].

Although the exogenous applications of ami/atasiRNAs or miRNA mimics have not been reported so far, we believe that these types of RNA molecules will be assessed and exploited in SIGS technology in the future.

### 3.5. RNA Guided CRISPR-Cas System

**Figure 4:**
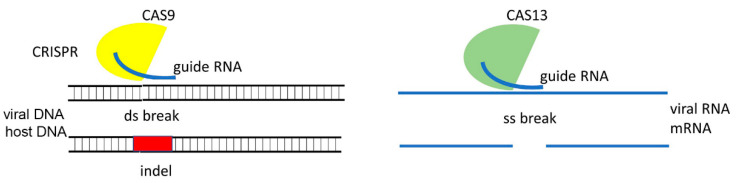
Schematic representation of the CRISPR-Cas technology. The Cas9 endonuclease in a complex with a sgRNA binds to the DNA target site and mediates a double-strand break which is then repaired, inducing deletions or insertions (gene knock-out). Cas13 in conjunction with sgRNA binds to the target RNA site and cleaves the RNA molecule.

As mentioned above, CRISPR-Cas technology has emerged as an expanding toolbox with which to generate antiviral resistance in various crop plants. In this regard, two of the most common approaches using CRISPR-Cas to control virus diseases is either by using it to directly target and inhibit virus machinery or by mutating “susceptibility” genes in host plants (reviewed in Kalinina et al. [14,15,16]. Most of the studies have been conducted using the Cas9 protein which is a DNA endonuclease [17], which via direction by a sgRNA, creates a break in the corresponding DNA target site which is then repaired by cellular machinery. This event leads to indels (insertions or deletions) in the ORF which often causes a target gene knock-out (Figure 4).

With regards to plant viruses, the CRISPR-Cas9 system was first deployed to combat DNA-containing geminiviruses. Using the transgenic expression of the CRISPR-Cas9 components (Cas9 and sgRNA), the designed sgRNAs were shown to be able to directly target various specific regions of the viral DNA genomes and consequently reduce or abolish disease symptoms caused by such geminiviruses as WDV [109]), tomato yellow leaf curl virus [107], beet curly top virus, and merremia mosaic virus [110], to name a few. CRISPR/Cas9 was also effective against cauliflower mosaic virus, another DNA containing virus (pararetrovirus) [111] (reviewed in [14,15,16]).

The discovery of CRISPR-related sgRNA-guided endonucleases specifically targeting RNA molecules such as FnCas9 and Cas13 [17], opened up new opportunities to control RNA-containing viruses in transgenic plants expressing FnCas9 or Cas13 and their sgRNAs. Successful examples of engineering RNA virus resistance using this approach include CMV [112], TMV [112], PVY [113], Southern rice black-streaked dwarf virus [114], rice stripe mosaic virus [114], and turnip mosaic virus [115].

These examples show that direct manipulation of viral DNA or RNA genomes by CRISPR-Cas may represent new technologies to engineer antiviral resistance. However, this approach requires persistent maintenance of the CRISPR-Cas components in the plant which thus far can only be carried through their transgenic expression. Due to GMO regulations, this may preclude or significantly limit practical applications of CRISPR antivirals.

To mitigate GMO-related barriers in engineering virus resistance, several other CRISPR-Cas approaches avoiding the use of GMO have been developed, which target host plant genes involved in regulating virus susceptibility. In the first approach, CRISPR-Cas components are initially expressed transgenically to modify a host “susceptibility” gene leading to virus resistance, and these transgenic components are then segregated out following sexual reproduction (reviewed in Kalinina et al. [14]). Although in the context of regulatory constraints this is a rather useful procedure, it is, however, a time-consuming and laborious process. This method is particularly unfeasible for clonally (vegetatively) propagated crops such as the potato. Additional technologies and approaches have started to evolve to circumvent many of these issues. For example, rather than transgenic expression, CRISPR-Cas components can be packaged into an RNP complex composed of the Cas protein and sgRNA [116], and these can be directly delivered into plant cells or tissues using different techniques, including protoplast transfection, biolistics, or vacuum infiltration [14]. We have recently developed a technique using chitosan microparticles as a platform for delivery of preformed Cas9-sgRNA complex into potato apical meristem using vacuum infiltration [117]. This approach completely excludes the use of foreign DNA and as such, is GMO-free. However, it still relies on a plant regeneration step, which may be awkward for many important crops or varieties.

The possibility of using the CRISPR-Cas system for editing virus susceptibility genes is at present also limited since there is little knowledge of potential host gene targets that influence virus susceptibility. Thus far, only two host gene groups (encoding eIF4s and coilin) which can affect virus susceptibility have been exploited for CRISPR editing to engineer virus resistance within a rather narrow range of viruses, such as potyviruses and rice tungro spherical virus [14,15,16,117,118,119]. As mentioned above, both *eIF4s* and coilin genes were previously used for the development of resistance in HIGS experiments (see above; [105,106]).

### 3.6. RNAi Versus CRISPR

As discussed, both RNAi and CRISPR-Cas have been successfully used as powerful tools for engineering plant virus resistance. However, the question arises as to which of these seemingly competing technologies is the right option for a particular application. There are several technical and methodological aspects to be addressed before making a choice:

*GMO.* As noted above, some CRISPR applications, in particular, direct targeting of plant viruses, require the permanent presence of CRISPR-Cas components in plants which is only possible in transgenic plants, whereas SIGS RNAi-based technology is transgene-free.

*Plant regeneration.* To inactivate host plant virus “susceptibility” genes, the constant presence of the Cas protein and sgRNA is not needed and hence, the use of transgenic plants can be avoided either by eliminating the Cas9/sgRNA transgenes or by delivering editing reagents as mRNAs or RNPs. However, the associated steps for the regeneration of whole plants from the edited cells with consequent identification of the edited lines are usually time-consuming, technically demanding, and costly. Moreover, many crop plants and varieties are recalcitrant to regeneration. So far, CRISPR transgene-independent techniques are only feasible within a limited range of plant species and varieties. In contrast, the RNAi-based SIGS approach does not require regeneration steps at all.

*Individual cultivars.* With CRISPR technology, each cultivar should be edited and tested independently whereas RNAi tools developed for targeting conserved gene sequences could be applicable to many cultivars.

*Time.* Preparation of RNAi tools (weeks) is significantly faster than production and selection of edited plants (months or years).

*Nature of phenotype*. Existing (transgene-free) CRISPR technologies induce permanent and irreversible gene edits in plants, whereas RNAi induces transient knockdown of gene expression. In many cases, transient perturbation of gene transcripts may be preferred, particularly when the complete knock-out of those genes that are essential could be lethal (or detrimental), but partial loss of them would not be. For example, virus “susceptibility” genes such as coilin genes (and others) possess many important cellular functions, and therefore their expression should be modulated in a temporary manner during virus attack only.

This analysis suggests that RNAi engineering of antiviral resistance may currently offer a number of significant advantages over CRISPR. While CRISPR technology has been a game-changer that led to remarkable progress in the development of new methods for targeted therapy of human diseases [120], the application of such medical-technical progress in the use of CRISPR technologies to crop improvement may not be straight-forward. For example, medical treatments involve correction of disease-causing genes/mutations in individual patients, whereas crop biotechnology operates with whole populations in the entire cultivar. With this in mind, the excellent potential of CRISPR technology in crop improvement applications is steadily progressing with the development of new approaches and the repurposing of existing technical aspects from medical fields. While the deployment of CRISPR in crop and plant science is becoming very popular as a tool for plant functional genomics and improvement, which in some cases rivals or exceeds RNAi technologies, RNAi approaches are still the main tool for targeting viral pathogens. However, for RNAi to become the method of choice, technical hurdles in the stability and efficient delivery of RNAi compounds into plant cells have to be overcome.

### 3.7. Stability and Uptake of RNA Molecules by Plant Cells

One of the major problems in topical applications of naked dsRNA molecules is their instability in the environment, which is typically manifested in a short antiviral protection window when applied against virus infections [26]. Therefore, RNA-based biocontrol compounds (such as dsRNAs, amiRNAs, or tasiRNAs) should be formulated for protection against degradation. An emerging method for the protection of RNA molecules is to use nano-protectors. An example is the loading of the virus-specific dsRNA on layered double hydroxide clay nanosheets which significantly increases the time of dsRNA action (from 5–7 days to 26 days [97]). Other proposed RNA protection platforms include small spherical cells lacking chromosomes that encapsulate dsRNA, liposomes, complexes with nanoparticles or protein carriers, DNA nanostructures, and artificial extracellular vesicles (reviewed in Taning et al. [25]).

Another concern is the capacity of RNAi compounds to penetrate cells, particularly where the large complexes which protect the RNA from degradation may significantly reduce permeability. Plants have evolved stringent security control systems (barriers) which govern specific signaling and transport pathways that prevent foreign molecules from penetrating and transporting through the plant tissues. The cuticle and cellulose cell wall are the first barriers typically encountered which are inhibitory to cell entry [121]. The next barrier encountered is plasmodesmata, which tightly regulate the flow of components at the cell-to-cell level, while vascular tissues such as phloem and xylem may act as barriers for long-distance transport. Indeed, the plant transport network is fully permeable only to some low-molecular-weight compounds, but specific chaperones able to increase the permeability of the control systems are used to allow entry of larger macromolecules or macromolecular complexes into and through this network [122]. For example, plant viruses comparable in size with nanoparticles may spread in a plant only if they encode compatible movement proteins able to modify plant transport systems [122]. Therefore, although exogenously derived small RNAs may mimic natural siRNAs and miRNAs that are able to move through the plant transport system, initial penetration and spread of the exogenous RNA complexes may be a bottleneck due to their structure and dimensions. A potential solution may also be found in nanotechnology, as an intriguing and currently unexplained attribute of some but not all nanoparticles are their ability to penetrate plant tissues and spread throughout the plant in spite of the strict “security” controls. Mesoporous silica [123] and chitosan [117] nanoparticles may be good examples illustrating great capacity as nanocarriers for the delivery of bioactive molecules. Thus, the identification and optimization of the most suited nanoplatforms for RNA delivery in plant cells is a key challenge for future research. There might be other factors affecting the efficiency of cell permeability such as the use of chemical surfactants, adjuvants, pH of carriers, timing or duration of treatment, and others, which also must be compatible with exogenous RNAi.

## 4. Conclusions and Perspectives

Various factors, such as climate change, decreases in agricultural land and global population growth as well as hurdles associated with GMO and current chemical pesticide regulations, have promoted the development of new biosafe and eco-friendly technologies for crop protection against pests and pathogens. Among them are RNA-based biocontrol technologies, such as RNAi and CRISPR. Although both of them are still in the research and development pipeline, they are well anticipated to be exploited for engineering plant virus resistance in agricultural practice. In spite of the popularity and great capabilities of the CRISPR-Cas system, RNAi approaches seem to be preferable at least for direct targeting plant viral pathogens. Commercial expectations surrounding RNAi applications are rising, with great interest towards sprayable RNA-based techniques from agribiotech giants such as Bayer Crop Science, Monsanto (now acquired by Bayer) and Syngenta, and many other newly developed start-up companies [26]. This trend is supported by the increased simplicity of production and decreased production costs of RNAi compounds [98]. Significant efforts are currently underway to expand and optimize the RNAi based toolbox for topical applications (dsRNAs, amiRNAs, tasiRNAs, miRNA mimics).

In addition, we are well aware that the emergence of CRISPR-Cas editing tools will significantly strengthen the arsenal of applications for genetic crop improvement. Unprecedented advances have been achieved by using CRISPR-Cas technology in basic plant biology. CRISRP-Cas has been proved to be a vital tool for plant functional genomics, including the generation of CRISPR/Cas mutant libraries for developing genome-wide mutation screens [124]. Another promising way to further improve the CRISPR-Cas technology may lie in the application of retrons. Retrons, or bacterial retroelements, encode reverse transcriptase and an ncRNA. The reverse transcriptase uses the ncRNA as a template, generating a chimeric RNA/DNA, with a natural function to cause the phage-infected cells to commit suicide (thus preventing further spread of infection) [125,126]. For biotechnological applications in eukaryotic organisms (including plants), the template for DNA synthesis in the retron can be replaced by any desired sequence and used, for example, in conjunction with the CRISPR elements to manipulate genes in various ways.

However, there are some common technical challenges and societal problems that must be addressed for both RNAi-SIGS and CRISPR-Cas technologies before they take a certain unique place in engineering plant virus resistance.

Identification of novel host susceptibility targets among coding or non-coding RNAs that should be inactivated or modified by RNAi or CRISPR would be a crucial next step to further develop basic engineering strategies for virus resistance. It could also be useful to expand a potential list of gene targets by identifying gene/ncRNA targets in genomes of insect vectors to prevent transmission of plant viruses. RNAi has recently been successfully used to control insect pests [26].

Another critical factor to be considered is the off-target effects of both RNAi and CRISPR technologies. Exogenously applied long dsRNAs can produce many different siRNA species with a higher chance of causing off-target host gene regulation [127]. However, the use of small amiRNAs and tasiRNAs could potentially minimize such off-target effects. It is also should be noted that unlike CRISPR-Cas9, the RNAi systems induce inheritable and temporary modifications of gene expression in plants and may be preferable to heritable off-target gene editing. Anyway, reducing the off-target effects for both the RNAi and CRISPR approaches becomes an important challenging task.

The rapid progress in RNAi and CRISPR applications also desperately requires the improvement of protective delivery platforms that allow efficient uptake and dissemination of RNA silencing or CRISPR editing components by plant cells. Cooperative efforts between plant biologists, geneticists, and nanotechnologists may provide a real game-changer in such crop protection technology.

Finally, clear and standardized regulatory guidelines are required for the development of non-transgenic externally applied RNAi and CRISPR tools to enable them to progress to agricultural markets.

## Figures and Tables

**Table 1:** Foliar application of exogenous dsRNAs for engineering plant virus resistance.

Virus/Viroid	RNA Production Method	Delivery Method	Host Plant/Crop	Reference
PMMoV, TEV, AMV	*In vitro* synthesized dsRNAs	Mechanical inoculation	*Nicotiana tabacum* *Capsicum chinense* (pepper), *Nicotiana benthamiana*	[85]
PMMoV, TMV, PPV	Bacterially expressed dsRNAs (crude extracts)	Mechanical inoculation or spraying	*N. benthamiana*	[86]
PSTVd, CEVd, CChMVd	*In vitro* synthesized dsRNAs	Mechanical inoculation	*Solanum lycopersicum* (tomato), *Gynura aurantiaca* (gynura), *Dendranthema grandiflora* (chrysanthemum)	[87]
TMV	Bacterially expressed dsRNA (crude extracts)	Mechanical inoculation	*N. tabacum*	[88]
PVY	Bacterially expressed hpRNAs (crude extracts)	Mechanical inoculation	*N. tabacum*	[89]
TMV, PVY	Bacterially expressed hpRNAs (crude extracts)	Mechanical inoculation	*N. tabacum*	[90]
SCMV	Bacterially expressed hpRNA (crude extracts)	Spraying	*Zea mays* (maize)	[91]
PRSV	Bacterially expressed hpRNA (crude extracts)	Mechanical inoculation	*Carica papaya* (papaya)	[92]
CymMV	Bacterially expressed dsRNAs and ssRNAs (crude extracts)	Mechanical inoculation	*Brassolaeliocattleya hybrida* (orchid)	[93]
PSbMV	*In vitro* synthesized dsRNA	Spraying	*Pisum sativum* (pea)	[94]
TMV	*In vitro* synthesized dsRNA	Mechanical inoculation	*N. tabacum*	[95]
CMV, PMMoV	*In vitro* synthesized dsRNAs or bacterially expressed dsRNAs (crude extracts); applied directly or loaded into LDH	Spraying	*N. tabacum, Vigna unguiculata* (cowpea)	[96]
ZYMV	*In vitro* synthesized dsRNAs	Mechanical inoculation	*Citrulus lanatus* (watermelon)*, Cucurbita pepo* (squash), *Cucumis sativus* (cucumber)	[97]
TMV	Bacterially expressed or in vitro synthesized dsRNA	Mechanical inoculation, spraying	*N. benthamiana*	[98]
BCMV	Chemically synthesized dsRNA applied directly or loaded into LDH	Spraying	*N. benthamiana, Vigna unguiculata* (cowpea)	[99]
ToLCV, CMV	*In vitro* synthesized dsRNAs	Mechanical inoculation	*S. lycopersicum* (tomato), *N. tabacum*	[100]
PRSV	*In vitro* synthesized dsRNA	Mechanical inoculation	*Carica papaya*	[101]

## Data Availability

Available data are presented in the manuscript.
